# Supplementary material for: WordCommentsAnalyzer: A windows software tool for qualitative research
Source: F1000Res. 2018 Sep 4;7:536. Originally published 2018 May 3. [Version 2] doi: 10.12688/f1000research.14819.2 (PMC6137411; doi:10.12688/f1000research.14819.2)
Supplement: Supplementary file 3 [file f1000research-7-17614-s0002.tgz › b2a79496-4ada-47c4-9682-a99ce867836d.zip!/Eight Iranian Health Journals/Salmand_Iranian Journal of Ageing _6.docx]

© Salmand_Iranian Journal of Ageing . This work is licensed under CC BY-NC 4.0 (https://creativecommons.org/licenses/by-nc/4.0/).

Volume & Issue: دوره 11 - شماره 4

Title: بررسی نگرش به مراقبت از سالمندان از دیدگاه دانشجویان پرستاری دانشکده پرستاری و مامایی استان کرمانشاه در سال 1394

http://salmandj.uswr.ac.ir/article-1-1001-fa.html

اهداف نگرش ارائه‌دهندگان خدمات سلامت به مددجویان می‌تواند عاملی مهم و زمینه‌ساز برای ارائه مراقبتی مؤثر و اثربخش باشد؛ به‌عبارت­‌دیگر درصورتی‌که پرستاران در ارتباط با مددجویان نگرشی مثبت داشته باشند، با انگیزه و عزمی راسخ‌تر می‌توانند به ارائه خدمات بالینی بپردازند. پژوهش حاضر با هدف تعیین نگرش کارآموزان و کاروزان پرستاری درزمینه مراقبت از سالمندان انجام شده است. مواد و روش ها مطالعه توصیفی‌تحلیلی حاضر روی 211 نفر از دانشجویان پرستاری، شامل 147 کارآموز و 64 کاروز دانشکده پرستاری و مامایی استان کرمانشاه در سال 1394 انجام شد. روش نمونه‌گیری کارآموزان به‌صورت تصادفی ساده و کارورزان به‌صورت سرشماری بود. ابزار جمع‌آوری داده‌ها پرسش‌نامه‌ای با 21 سؤال بود. داده‌ها با نسخه 22 نرم‌افزار SPSS و آزمون تی مستقل تجزیه‌وتحلیل شد. یافته ها میانگین نگرش سالمندی در کارآموزان 97/5±63/72 و کارورزان 27/7±6/74 بود که ازنظر آماری اختلاف معناداری وجود داشت (05/0P<). بیشتر کارآموزان (89/8درصد) و کارورزان (81/3درصد) درزمینه مراقبت از سالمندان نگرش متوسطی داشتند. گویه «پرستاری از سالمندان اتلاف‌وقت است؛ زیرا آن‌ها به پایان عمر خود رسیده‌اند»، از دیدگاه کارآموزان 57/0±67/4 و کارورزان 47/0±73/4 بیشترین میانگین را در بین گویه‌ها داشت. بین این دو میانگین، اختلاف معناداری وجود نداشت (05/0P>). نتیجه گیری در دانشجویان سال بالا، نگرش مثبت به مراقبت از سالمندان افزایش یافته است. به‌نظر می‌رسد آموزش مراقبت از سالمند در ایجاد این نگرش مؤثر بوده است. باتوجه‌به اینکه بیشتر دانشجویان به سالمندان نگرشی متوسط (نه خوب) داشتند، به‌کارگیری آموزش‌های تخصصی‌تر برای ارتقای سطح دانش و به‌تناسب آن نگرش سالمندی ضرورت دارد.

Title: بررسی ارتباط ناتوانی‌های بینایی، شنوایی، حافظه و قدرت چنگش دست با میزان قابلیت استفاده سیستم‌های دردسترس سالمندان مقیم خانه‌های سالمندان شهرکرمان

http://salmandj.uswr.ac.ir/article-1-1090-fa.html

اهداف براساس تعریف سازمان جهانی بهداشت، به افراد 60‌ساله یا بیش‌ازآن سالمند گفته می‌شود. سالمندی بخشی از فرایند زیستی است که تمام موجودات زنده ازجمله انسان را دربر‌می‌گیرد. افزون‌براین قسمتی از زندگی است که پس از میان‌سالی رخ می‌دهد و همواره با مشکلات و ناراحتی‌ها و کاهش توان فیزیکی افراد در انجام کارها همراه است. در فرایند سالمندی سیستم‌های مختلف بدن تحلیل می‌روند. همچنین با کاهش توانایی‌ در سالمندان وابستگی به دیگران ایجاد می شود. این مطالعه باهدف بررسی ارتباط ناتوانی‌های بینایی، شنوایی، حافظه و قدرت چنگش دست با میزان قابلیت استفاده سیستم‌های دردسترس سالمندان مقیم خانه‌های سالمندان شهرکرمان انجام شد. مواد و روش ها این مطالعه مقطعی روی 110 نفر (58 مرد و 52 زن) سالمند مقیم خانه‌های سالمندان شهر کرمان انجام شد. جمع‌آوری اطلاعات به‌صورت سرشماری بود و تمام داده‌های مدنظر با مراجعه حضوری به خانه‌های سالمندان و دیدار چهره‌به‌چهره با افراد در ساعاتی از روز جمع‌آوری شود که تداخلی با دیگر فرایندهای جاری کارکنان خدماتیِ خانه‌های سالمندان نداشته باشد. به‌منظور بررسی ناتوانی‌های شنوایی، حافظه و بینایی به ترتیب از روش ادیومتری، پرسش‌نامه استاندارد سنجش حافظه و روش بینایی‌سنجی چارت E استفاده شد. از دینامومتر جامار به‌منظور اندازه‌گیری نیروی چنگش قدرتی دست‌ها استفاده شد. قابلیت استفاده از سیستم‌ها (تلویزیون، رادیو و یخچال) با استفاده از پرسش‌نامه SUS در مقیاس لیکرت سنجیده شد. پس از کسب اطلاعات لازم داده‌های جمع‌آوری شده با استفاده از نسخه شانزدهم نرم‌افزار SPSS و آزمون‌های آماری در سطح معنی‌داری 05/0 تجزیه‌وتحلیل شد. یافته ها محدوده سنی افراد بین 60 تا 96 بود. میانگین سنی مردان 70/73 و زنان 19/77 و میانگین سنی کل جامعه 35/75 به‌دست آمد. بیشترین تعداد مردان سالمند با 27 نفر (4/46درصد) در گروه سنی 60 تا 70 و در زنان سالمند با 17 نفر (2/32درصد) در گروه سنی 80 تا 90 وجود دارد. همچنین کمترین فراوانی در هر دو جنس در گروه سنی 90 تا 100 (مردان 9/6درصد و زنان 8/3درصد) وجود دارد. میانگین سنی جامعه آماری 35/75 به‌دست آمد. تفاوت ناتوانی شنوایی و بینایی در گوش و چشم چپ سالمندان معنادار بود. از لحاظ ناتوانی حافظه‌ای تفاوت معناداری بین زنان و مردان مشاهده نشد. اما تفاوت نیروی چنگش قدرتی دست‌ها درمردان و زنان معنادار بود (P=0/0001). بیشترین میزان قابلیت استفاده در هر دو گروه مردان و زنان در ارتباط با یخچال و کمترین این مقدار در ارتباط با تلویزیون به‌دست آمد. ناتوانی‌های بررسی‌شده و قدرت چنگش دست در سالمندان با قابلیت استفاده از هر سه وسیله تلویزیون، یخچال و رادیو ارتباط معناداری داشت.  نتیجه گیری با افزایش سن بر میزان ناتوانی‌های سالمندان افزوده می شود و قابلیت استفاده از سیستم‌ها کاهش می یابد؛ بنابراین لازم است برای افزایش قابلیت استفاده از سیستم‌های دردسترس سالمندان از کاهش توانایی آن‌ها جلوگیری شود.

Title: بررسی رابطه بین حمایت اجتماعی و پایبندی به مذهب با اضطراب مرگ در سالمندان

http://salmandj.uswr.ac.ir/article-1-1311-fa.html

اهداف سالمندی فرایندی زیستی است که تمام موجودات زنده ازجمله انسان آن را تجربه می‌کنند. از موضوعات مهم در این دوره، نزدیکی زمانی با مرگ و اضطراب ناشی از آن است. هدف از پژوهش حاضر بررسی رابطه بین مؤلفه‌های حمایت اجتماعی و پایبندی به مذهب با اضطراب مرگ در سالمندان زن و مرد، باتوجه‌به وضعیت تأهل بود. مواد و روش ها در این پژوهش از روش همبستگی و علّی مقایسه‌ای استفاده شد. در این پژوهش 376 نفر نمونه شامل 190 نفر زن و 186 نفر مرد (مجرد و متأهل)  بررسی شدند. نمونه‌ها از بین شهروندان با مدرک دیپلم و دامنه سنی 60 تا 75 سال از مناطق 2، 3، 5، 6 و 22 شهر تهران که مقیم آسایشگاه نبودند، به‌صورت دردسترس انتخاب شدند. برای سنجش متغیرهای پژوهش از مقیاس‌های اضطراب مرگ (فرم 27 سؤالی) و حمایت اجتماعی (فرم 19 سؤالی) و نگرش‌های مذهبی (فرم 26سؤالی) استفاده شد. داده‌ها با روش تحلیل واریانس یک‌طرفه و آزمون پیرسون و با استفاده از نسخه 21 نرم‌افزار SPSS تحلیل شدند. یافته ها نتایج محاسبه همبستگی نشان داد از چهار مؤلفه حمایت اجتماعی، بین دو مؤلفه اضطرب مرگ یعنی ترس از عواقب مردن و (P<0/05) ترس از مرگ توسط دیگران (P<0/05) با اضطراب مرگ کلی (P<0/01) رابطه معنادار مثبت وجود دارد. علاوه‌براین بین پایبندی به مذهب با اضطراب مرگ، صرفاً بین مؤلفه اعتقادی با مؤلفه ترس از عواقب مُردن رابطه معنادار مثبت وجود دارد (P<0/01). همچنین نتایج تحلیل واریانس نشان داد در میان مردان مجرد و متأهل درزمینه اضطراب مرگ تفاوت معناداری وجود دارد؛ به‌طوری‌که مردان مجرد اضطراب مرگ بیشتری را تجربه می‌کنند (P<0/01). همچنین میانگین نمره‌های اضطراب مرگ در زنان بیشتر از مردان است (P<0/01)؛ اما بین میزان اضطراب مرگ زنان مجرد و متأهل تفاوت معناداری وجود ندارد. نتیجه گیری افزایش تعامل اجتماعی، تقویت پایبندی به مذهب و متأهل‌بودن موجب کاهش اضطراب مرگ می‌شود که عاملی شایع و مختل‌کننده بهداشت روانی سالمندان به‌ویژه زنان سالمند است.

Title: تحلیل تجربه سالمندان ساکن در اجتماع از فعال‌بودن: مطالعه کیفی استراتژی‌های سالمندی فعال

http://salmandj.uswr.ac.ir/article-1-1142-fa.html

اهداف در دهه‌های اخیر لزوم برخورد مناسب و جامع با مسئله سالمندی جمعیت که تضمین‌کننده کیفیت زندگی آن‌ها باشد منجر به روی‌آوردن جوامع به دیدگاه مثبت به سالمندی و به‌ویژه سالمندی فعال شده است. درک بومی از سالمندی ضرورتی انکارناپذیر است. از این‌رو پژوهش حاضر با هدف تحلیل استراتژی‌های سالمندی فعال بر اساس تجربه سالمندان ساکن در اجتماع صورت گرفت. مواد و روش ها این مطالعه از نوع کیفی بود که به روش نظریه مبنایی در سال 1393-1394 انجام شد. در این پژوهش از روش نمونه‌گیری هدفمند و نظری استفاده شد. در این مطالعه با مراجعه به فرهنگسراها، کانون‌های فرهنگی و ادبی و پارک‌های شهر تهران با 35 سالمند در دامنه سنی 61 تا 85 سال مصاحبه‌های نیمه ساختاریافته انجام شد تا داده‌ها به مرحله اشباع برسند. داده‌ها با روش استراوس و کوربین تجزیه‌وتحلیل شد. اعتبار داده‌ها بر اساس معیارهای پیشنهادی اسکواند و لینکن و گوبا تأیید شد. یافته ها بر اساس تجارب سالمندان استراتژی‌های فعال‌بودن شامل 41 مفهوم، 7 زیرمقوله و 5 مقوله عمده است. مقوله‌های عمده و زیرمقوله‌های آن‌ها عبارتند از: استراتژی‌های اولیه انزواگریزی (شامل عدم خانه‌نشینی و تعامل‌گرایی)، مشارکت اجتماعی (شامل تداوم نقش‌آفرینی حرفه‌ای، فعالیت‌های داوطلبانه و مشارکت اجتماعی و نهادی)، استراتژی‌های نگرشی و یادگیری (شامل بینش فعال و یادگیری)، پویایی جسمی و مدیریت امور و اوقات خانه‌نشینی. نتیجه گیری سالمندی فعال در گرو استراتژی‌هایی است که در سه حوزه بینش سالمند و محدوده زندگی (خانه) و محیط اجتماع تحقق می‌یابد. بنابراین برای داشتن سالمندانی فعال، متخصصان و برنامه‌ریزان باید به این سه حوزه توجه کنند.

Title: ارتباط خودکارآمدی و کیفیت زندگی سالمندان مقیم آسایشگاه‌های شهر تهران

http://salmandj.uswr.ac.ir/article-1-1170-fa.html

اهداف خودکارآمدی متغیر مهمی در حوزه سالمندی است که کمتر به آن توجه شده است. کیفیت زندگی یکی از مهم‌ترین شاخص‌های توسعه انسانی است. این مطالعه با هدف بررسی ارتباط بین خودکارآمدی و کیفیت زندگی سالمندان مقیم آسایشگاه‌های شهر تهران در سال 1394 انجام شد. مواد و روش ها در این مطالعه توصیفی و تحلیلی که به روش مقطعی انجام شد، جامعه آماری شامل تمام سالمندان مقیم آسایشگاه‌های سالمندی شهر تهران بود. 210 نفر از سالمندان مقیم آسایشگاه‌های شهر تهران با توجه به معیارهای پژوهش انتخاب شدند. نمونه‌گیری با روش سهمیه‌ای انجام شد و از هر آسایشگاه به نسبت تعداد سالمندان مقیم نمونه‌گیری تصادفی ساده به عمل آمد. به منظور جمع‌آوری داده‌ها از مقیاس خودکارآمدی عمومی و پرسش‌نامه کیفیت زندگی سالمندان لیپاد و پرسش‌نامه جمعیت‌شناختی استفاده شد. ابزارها از نظر پایایی ارزیابی شدند. میانگین سن سالمندان 78/11±66/78 سال بود. داده‌ها بعد از جمع‌آوری به نسخه 18 نرم‌افزار SPSS وارد شد و با آمار توصیفی شامل فراوانی و میانگین و انحراف‌معیار و آمار استباطی شامل آزمون کای اسکوئر، ضریب همبستگی پیرسون، آزمون کلموگروف اسمیرنوف، تحلیل واریانس یک‌طرفه و آزمون تی مستقل تجزیه‌وتحلیل شد. یافته ها میانگین خودکارآمدی وکیفیت زندگی سالمندان مقیم آسایشگاه‌های سالمندی شهر تهران به ترتیب 00/4±68/17 و 80/9±88/27 بود. بین سن و جنسیت و وضعیت تأهل با خودکارآمدی در سالمندان رابطه معنادار وجود ندارد. بین سطح تحصیلات (042/0P=/) با خودکارآمدی تفاوت معنادار وجود دارد. بین سن (047/0P=) و سطح تحصیلات (038/0P=) با کیفیت زندگی ارتباط معنادار وجود دارد. بین جنسیت و وضعیت تأهل با کیفیت زندگی ارتباط معنادار وجود ندارد. نتیجه گیری با توجه به نتایج این مطالعه، خودکارآمدی و کیفیت زندگی سالمندان مقیم آسایشگاه‌های سالمندی شهر تهران پایین بود. با افزایش نمره خودکارآمدی در بین سالمندان مقیم سراهای سالمندی،کیفیت زندگی آنان نیز افزایش می‌یابد.

Title: تأثیر طول پیش‌دوره و دست برتری بر زمان واکنش ساده و انتخابی سالمندان

http://salmandj.uswr.ac.ir/article-1-1177-fa.html

اهداف با افزایش سن در عملکرد شناختی نظیر زمان واکنش تغییراتی رخ می‌دهد. با توجه به اهمیت آماده‌سازی در پاسخ به زمان واکنش در موقعیت‌های گوناگون زندگی، هدف از انجام این تحقیق بررسی تأثیر پیش‌دوره‌های مختلف 5/0، 5/1، 5/2 و 5/3 ثانیه و دست برتری بر زمان واکنش شنیداری ساده و انتخابی است. مواد و روش ها این تحقیق از نوع نیمه‌تجربی است که با نرم‌افزار محقق‌ساخته که روایی و پایایی آن تأیید شده است انجام گرفت. جامعه آماری این تحقیق شامل تمام سالمندان شهر اهواز بود که از بین آن‌ها 30 نفر به صورت نمونه‌گیری هدفمند انتخاب شدند و در دو گروه دست برتری چپ و راست قرار گرفتند. آزمون زمان واکنش به صورت ساده و انتخابی با پیش‌دوره‌های مختلف به صورت متغیر از هر فرد گرفته شد. داده‌ها با استفاده از تحلیل واریانس درون‌گروهی تجزیه‌وتحلیل شد و سطح معناداری 05/0>P در نظر گرفته شد. یافته ها نتایج نشان داد بین پیش‌دوره‌های مختلف و نیز بین زمان واکنش ساده و انتخابی تفاوت معناداری وجود دارد (05/0>P). پیش‌دوره‌های خیلی کوتاه (5/0 و 5/1) باعث افزایش زمان واکنش می‌شود. همچنین مشخص شد افراد چپ‌دست زمان واکنش کوتاه‌تری نسبت به افراد راست‌دست دارند. نتیجه گیری به دلیل اینکه فرایندهای پردازش شناختی در سالمندان کندتر صورت می‌گیرد، به منظور آماده‌سازی افراد سالمند برای اجرای مهارت‌های مختلف بهتر است از پیش‌دوره‌های خیلی کوتاه اجتناب کرد. همچنین احتمالاً افراد چپ‌دست به دلیل برتری عصبی ذاتی ناشی از حجم ماده شیار مرکزی خاکستری مغز و درگیری بیشتر نیم‌کره راست مغز که رشته‌های عصبی بیشتری دارد زمان واکنش کوتاه‌تری نسبت به افراد راست‌دست دارند.

Title: ارتباط بین بهزیستی روان‌شناختی و استحکام روانی در سالمندان: نقش میانجی فعالیت بدنی

http://salmandj.uswr.ac.ir/article-1-1183-fa.html

اهداف در سال‌های اخیر مسائل روان‌شناسی و روان‌درمانی به‌خصوص در سالمندان مورد توجه قرارگرفته است. به دلیل افزایش آمار اختلالات روانی در سالمندان، شناسایی عوامل تأثیرگذار بر سلامت روان سالمندان اهمیت پیدا کرده است. هدف از انجام این تحقیق بررسی ارتباط بین بهزیستی روان‌شناختی و استحکام روانی در سالمندان با نقش میانجیگر فعالیت بدنی است.  مواد و روش ها این پژوهش از نوع توصیفی همبستگی است که داده‌های آن با استفاده از پرسش‌نامه‌های بین‌المللی فعالیت بدنی، استحکام روانی و بهزیستی روان‌شناختی از بین سالمندان جمع‌آوری شد. بدین منظور 217 نفر (107 سالمند فعال و 110 سالمند غیرفعال) با استفاده از روش نمونه‌گیری هدفمند با میانگین سنی 12/3±38/65 انتخاب شدند و همه آن‌ها پرسش‌نامه‌های مذکور را تکمیل کردند. هوشیاری کامل و داشتن سلامت روانی و ذهنی از معیارهای ورود به مطالعه بود. داده‌ها با استفاده از ضریب همبستگی بررسی شد. برای تعیین نقش واسطه‌ای فعالیت بدنی از آزمون بوت‌استرپ و برای تجزیه‌وتحلیل داده‌ها از نسخه 21 نرم‌افزار SPSS و نسخه 18 نرم‌افزار AMOS استفاده شد. سطح معناداری 05/0>P در نظر گرفته شد. یافته ها شرکت‌کنندگان با استفاده از پرسش‌نامه بین‌المللی فعالیت بدنی در دو گروه فعال و غیرفعال قرار گرفتند. نتایج نشان داد بین اکثر خرده‌مقیاس‌های استحکام روانی و بهزیستی روان‌شناختی ارتباط مثبت و معناداری وجود دارد. همچنین مشخص شد میانگین استحکام روانی و بهزیستی روان‌شناختی سالمندان فعال نسبت به سالمندان غیرفعال بالاتر است و نیز متغیر فعالیت بدنی توانست به عنوان عامل میانجیگر بین استحکام روانی و بهزیستی روان‌شناختی ایفای نقش کند. نتیجه گیری با توجه به نتایج پژوهش حاضر و نقش میانجی فعالیت بدنی در استحکام روانی و بهزیستی روان‌شناختی، تشویق سالمندان به انجام فعالیت‌های بدنی و ورزشی می‌تواند به افزایش سلامت روانی و افزایش بهزیستی روان‌شناختی در آن‌ها کمک کند.

Title: تأثیر تمرین‌های نوروفیدبک بر بهبود زمان واکنش مردان سالمند غیرفعال

http://salmandj.uswr.ac.ir/article-1-1221-fa.html

اهداف با افزایش سن زمان واکنش افراد در اثر ناتوانی در پیش‌بینی درست محرک افزایش خواهد یافت که این عمل شناختی با ورود به مرحله سالمندی شدیدتر می‌شود. برنامه نوروفیدبک از طریق تنظیم ناهنجاری‌های امواج مغزی به صورت خودتنظیمی، می‌تواند عملکرد شناختی افراد را تحت تأثیر قرار دهد. هدف از انجام این تحقیق بررسی تأثیر تمرین‌های نوروفیدبک بر بهبود زمان واکنش سالمندان مرد غیرفعال است. مواد و روش ها این تحقیق از نوع نیمه‌تجربی است. تعداد 18 نفر مرد سالمند با میانگین سنی (12/3±38/65) انتخاب و به‌طور مساوی و کاملاً تصادفی در دو گروه آزمایش و کنترل قرار گرفتند. به منظور ارزیابی زمان واکنش شرکت‌کنندگان از دستگاه زمان واکنش YB-1000 استفاده شد. هر دو گروه در سه مرحله (پیش‌آزمون و آزمون میانی و پس‌آزمون) آزموده شدند. آزمودنی‌های گروه تجربی دوازده جلسه در تمرین‌های نوروفیدبک شامل افزایش توان بتا و کاهش توان تتا شرکت کردند. به منظور حذف اثر تلقین، گروه کنترل نیز در همان شرایط قرار گرفت، اما فقط امواج ضبط‌شده تمرین‌های گروه تجربی را به عنوان تمرین مشاهده کردند. یافته ها نتایج حاصل از آزمون تحلیل عاملی کوواریانس نشان داد در قیاس با گروه کنترل، تمرین‌های نوروفیدبک (افزایش موج بتا و کاهش موج تتا) موجب بهبود معنادار (05/0>P) در عملکرد زمان واکنش گروه آزمایش شد. نتیجه گیری تمرین‌های نوروفیدبک، با استفاده از بازداری یا تقویت فعالیت امواج مغزی و همچنین بهبودی در استفاده مفید از حافظه می‌تواند به عنوان یک روش تمرینی مؤثر در بهبود پردازش‌های شناختی نظیر زمان واکنش سالمندان مدنظر قرار گیرد.

Title: خودکارآمدی و متغیرهای جمعیت‌شناختی مرتبط با آن در سالمندان فریدون‌شهر

http://salmandj.uswr.ac.ir/article-1-1198-fa.html

اهداف خودکارآمدی در تمام رده‌های سنی به‌ویژه درسالمندان به لحاظ شرایط سنی و تغییرات فیزیولوژیک و آسیب‌پذیری آن‌ها بحث وسیعی را بین متخصصان ایجادکرده است. بسیاری از مطالعات، خودکارآمدی را مهم‌ترین عامل پیش‌بینی‌کننده رفتارهای بهداشتی بزرگ‌سالان و سالمندان می‌دانند. این مطالعه با هدف بررسی خودکارآمدی در سالمندان شهر فریدون‌شهر انجام شد. مواد و روش ها مطالعه حاضر از نوع توصیفی تحلیلی و مقطعی بود. بدین منظور 200 نفر از سالمندان فریدون‌شهر به روش نمونه‌گیری سیمستاتیک تصادفی انتخاب شدند. میانگین سنی سالمندان 3/7±02/68 بود. 101 نفر (5/50درصد) زن و 99 نفر (5/49) مرد بودند. برای گردآوری داده‌ها از پرسش‌نامه‌ای متشکل از دو بخش استفاده شد. بخش اول شامل اطلاعات جمعیت‌شناختی سالمندان و بخش دوم شامل پرسش‌نامه خودکارآمدی شرر بود. برای تجزیه‌وتحلیل داده‌ها از نسخه 20 نرم‌افزار SPSS و آزمون‌های آماری توصیفی اسپیرمن و آزمون تی مستقل و همبستگی پیرسون استفاده شد.  یافته ها میانگین نمره خودکارآمدی سالمندان 6/26 با انحراف‌معیار 8/6 به دست آمد. آزمون تی مستقل نشان داد در سالمندانی که بیماری مزمن داشتند نمره خودکارآمدی به‌طور معناداری کمتر از سالمندانی بود که بیماری مزمن نداشتند. همچنین میانگین نمره خودکارآمدی در سالمندانی که باخانواده زندگی می‌کردند به‌طور معناداری بیشتر از سالمندانی بود که تنها زندگی می‌کردند. میانگین نمره خودکارآمدی در سالمندان شاغل به صورت معناداری بیشتر از سالمندان بیکار بود.  نتیجه گیری خودکارآمدی یکی از اجزای مهم تغییرات سبک زندگی است که می‌تواند در تغییر رفتار و ایجاد رفتار بهداشتی مؤثر باشد. در این مطالعه خودکارآمدی سالمندان در حد متوسط بود. لذا توصیه می‌شود به منظور بالابردن و ارتقای خودکارآمدی در سالمندان مداخلات آموزشی و ارتقای سلامت طراحی و اجرا شود.

Title: مطالعه مروری: چالش‌های سالمندان دیالیزی با تأکید بر سندروم‌های سالمندی

http://salmandj.uswr.ac.ir/article-1-1211-fa.html

اهداف دیالیز روش درمانی مناسبی برای سالمندان با نارسایی مزمن کلیه است؛ اما در این روش درمانی بیمار با مشکلات زیادی روبه‌رو می‌شود. مطالعه حاضر با هدف بررسی چالش‌های سالمندان دیالیزی با تأکید بر سندروم‌های سالمندی و شناخت بیشتر آن و پیشگیری است. مواد و روش ها در این مطالعه برای دستیابی به اهداف مطالعه، جست‌وجوی منابع الکترونیکی در پایگاه داده‌های اطلاعاتی گوگل‌اسکالر، پاب‌مد، اسکوپوس، پرکوست، ایران‌مدکس، الزویر و آی‌اس‌آی، با کلید واژگان انگلیسی و فارسی سالمندان، چالش، سندروم‌های سالمندی و همودیالیز بدون محدودیت زمانی تا پایان سال 2015 انجام شد. یافته ها سالمندان دیالیزی مشکلات جسمی و روحی‌وروانی زیادی دارند و میزان همبودی بیماری‌ها و مصرف دارو در آن‌ها در مقایسه با جوانان بیشتر است. این امر موجب می‌شود تا کیفیت زندگی پایین‌تری در مقایسه با دیگر گروه‌های سنی داشته باشند. سالمندان دیالیزی مشکلات بیشتری ازجمله سندروم‌های سالمندی شامل سقوط، ناتوانی در عملکرد، اختلال شناختی و آسیب‌پذیری را در مقایسه با سالمندان سالم تجربه می‌کنند. میزان شیوع این اختلالات در سالمندان دیالیزی بیشتر است و موجب نبودِ استقلال و ناتوانی و مرگ‌ومیر بیشتر می‌شود. نتیجه گیری با افزایش تعداد سالمندان دیالیزی، شناخت دقیق مشکلات و اختلالات این گروه سنی ضروری به‌نظر می‌رسد. با افزایش آگاهی و دانش می‌توان برای مراقبت و توان‌بخشی آنان برنامه‌ریزی دقیقی کرد.
